# Investigating the necessity of preoperative coronary angiography for infection-related cardiac implantable electronic device explantation

**DOI:** 10.1016/j.ahjo.2026.100762

**Published:** 2026-03-18

**Authors:** Tulio Caldonazo, Holly Scheler, Johannes Fischer, Hristo Kirov, Murat Mukharyamov, Stephanie Gräger, Angelique Runkel, Sebastian Reinartz, Mahmoud Diab, Torsten Doenst

**Affiliations:** a Department of Cardiothoracic Surgery, Friedrich-Schiller-University, Jena, Germany; b Department of Diagnostic and Interventional Radiology, University Hospital, Jena, Germany; c Department of Cardiac Surgery, Helios University Hospital Wuppertal, University Witten/Herdecke, Witten, Germany

**Keywords:** Cardiac implantable electronic device explantation, Infective endocarditis, Coronary angiography

## Abstract

**Background:**

Device-associated endocarditis is a potentially life-threatening condition that typically requires the removal of the cardiac implantable electronic device (CIED). The role of routine coronary angiography (CAG) as part of preoperative evaluation remains uncertain.

**Objectives:**

This study aims to assess the necessity of preoperative CAG and its impact on clinical outcomes in patients who underwent isolated CIED explantation due to infection.

**Methods:**

A single-center retrospective analysis was conducted at Jena University Hospital between 2007 and 2023. The primary outcome was 30-day mortality. The secondary outcomes were major perioperative complications. The data were displayed using descriptive statistics and classic 2-sided tests.

**Results:**

A total of 287 high-risk patients underwent isolated CIED explantation due to infection, of whom 120 underwent a preoperative CAG while 167 did not. Preoperatively, almost the half of the patients did not present history of coronary artery disease (No CAG: 53.9% and CAG: 45.0%), and the CAG group presented higher rates of lead vegetation (65.0% vs 52.7%, *p* = 0.04). Preoperative CAG had no significant effect on 30-day mortality (9.2% vs 9.6%, *p* = 1.00, mostly due to sepsis). Additionally, there was no significant difference in postoperative complications between the groups, including myocardial infarction (*p* = 1.00), bleeding (p = 1.00), acute renal failure (*p* = 0.76), or surgical conversion (*p* = 0.29).

**Conclusions:**

The analysis suggests that preoperative CAG does not significantly influence short-term outcomes after CIED explantation due to infection in our patient population. Systematic preoperative CAG may not be necessary for all patients. Moreover, non-invasive imaging modalities may have emerging importance in scenarios like this.

## 1 Introduction

Device-associated endocarditis is a serious, potentially life-threatening condition that often requires the removal of the cardiac implantable electronic device (CIED) [1], [2]. This infection can lead to complications such as septicemia, heart failure, or embolic events [3], [4], [5]. The treatment typically involves both antibiotic therapy and, in most cases, the removal of the CIED to prevent further systemic infection [6], [7].

Since removal of CIED‑leads can result in the need for emergently opening the sternum, exposing the heart and the potential use of cardiopulmonary bypass, most centers routinely perform a coronary angiograms (CAG) before the operation [8], [9]. CIED patients are often comorbid and carry an elevated risk of potentially asymptomatic but relevant coronary artery disease (CAD), which may complicate coming-off bypass in an emergency setting. However, the number of emergencies in CIED explantations is low and the management of such complications has never been thoroughly evaluated. We therefore assessed our experience with patients who underwent isolated CIED explantation due to infection in relation to the presence or absence of a preoperative CAG.

## 2 Methods

### 2.1 Study design

This is a single-center cohort retrospective observational study in patients with CIED infection who underwent isolated CIED explantation in our center between 2007 and 2023. The primary reasons why patients did not receive CAG were the surgeon's decision and alternative diagnostic strategies. The study was approved by our Ethical Committee (Nr. 2023-3020) and was conducted according to the Helsinki Declaration. Data from the patients were retrieved from clinical records. The patient consent was present and written during hospital admission. The dataset included detailed patient information relevant to their surgical and medical history.

### 2.2 Study outcomes

The primary outcome was 30-day mortality. The secondary outcomes were major perioperative complications: cerebrovascular event, vascular rupture, gastrointestinal complications, pericardial effusion, pleural effusion, pneumothorax as well as rhythm at discharge.

### 2.3 Statistical analysis

Continuous variables were reported using median and interquartile range (IQR) in the baseline demographics and as mean and standard deviation in the endpoint's description. Categorical variables were summarized by absolute and relative frequencies. Fisher exact test, Wilcoxon Mann-Whitney test, and Student's *t*-test were applied to compare binary and continuous variables. The null hypothesis was rejected if the *p* value related was ≤0.05.

## 3 Results

### 3.1 Study population

The entire population comprised 287 patients who underwent isolated CIED explantation due to infection, of whom 120 underwent a preoperative CAG while 167 did not. Table 1 shows the baseline characteristics of the overall population. There was no significant difference in the average age between the groups (69.2 ± 0.9 years for non-CAG vs. 73.2 ± 0.9 years for CAG, *p* = 0.53) or in the proportion of male patients (77.8% in non-CAG vs. 72.5% in CAG, *p* = 0.33).

**Table 1:** Demographics characteristics.

	No angiography *n* = 167	Angiography *n* = 120	*p*-Value
Age (years)	69.2 ± 0.9	73.2 ± 0.9	0.53
Male	130 (77.8)	87 (72.5)	0.33
Body-mass-index	27.2 ± 0.4	28.2 ± 0.4	0.12
Clinical signs			0.43

Clinical presentation			
Pacemaker pocket infection	78 (46.7)	47 (39.2)	
Fever	38 (22.8)	37 (20.8)	
Fatigue	5 (3.0)	3 (2.5)	
Poor appetite	1 (0.6)	0 (0)	
Weight loss	2 (1.2)	1 (0.8)	
Neurological symptoms	4 (2.4)	3 (2.5)	
Dyspnea	17 (10.2)	9 (7.5)	
Muscle and joint symptoms	1 (0.6)	1 (0.8)	
Cough	1 (0.6)	1 (0.8)	
Low back pain	0 (0)	2 (1.7)	
Chest pain	4 (2.4)	1 (0.8)	
Arrhythmia	2 (1.2)	0 (0)	
Skin lesions	0 (0)	0 (0)	
Sepsis	2 (1.2)	3 (2.5)	
EuroSCORE II	13.3 ± 15.5	12.7 ± 13.4	0.76
LVEF (%)	43.6 ± 18.0	47.9 ± 17.7	0.05
Lead vegetation	88 (52.7)	78 (65.0)	0.04
Largest vegetation (mm)	15.5 ± 8.9	13.9 ± 7.9	0.33
Smoking status			0.07
Smoked in the last 2 months	14 (8.4)	4 (3.3)	
Never smoked	58 (34.7)	45 (37.5)	
Ex-smoker	45 (26.9)	22 (18.3)	
Daily smoker	5 (3.0)	2 (1.7)	
Hyperlipidemia			0.40
Treated	100 (59.9)	77 (64.2)	
Untreated	5 (3.0)	1 (0.8)	
Hypertension			0.11
Treated	139 (83.2)	96 (80.0)	
Untreated	1 (0.6)	2 (1.7)	
Rhythm at admission			0.69
Sinus rhythm	59 (35.3)	46 (38.3)	
Atrial fibrillation	47 (28.1)	36 (30.0)	
Other rhythm	61 (36.5)	38 (31.7)	
Pre-operative ventilation	9 (5.39)	5 (4.2)	0.78
Vitamin K antagonist intake	38 (22.8)	22 (18.3)	0.38
COPD			0.77
With long-term medication	25 (15.0)	19 (15.8)	
Without long-term medication	8 (4.8)	4 (3.3)	
Peripheral arterial disease	22 (13.2)	19 (15.8)	0.61
Previous CVA	22 (13.2)	14 (11.7)	0.25
Antibiotics intake at admission	98 (58.7)	72 (60.0)	0.90
Previous PCI	46 (27.9)	35 (29.2)	0.89
Pre-operative RRT	19 (11.4)	22 (18.3)	0.26
Diabetes			0.42
Diet-controlled diabetes	15 (9.0)	13 (10.8)	
Oral antidiabetics	22 (13.2)	15 (12.5)	
Insulin + oral medications	9 (5.4)	2 (1.7)	
Insulin-dependent diabetes	35 (21.0)	32 (26.7)	
Surgery urgency			0.17
Elective	29 (17.4)	18 (15.0)	
Urgent	99 (59.3)	60 (50.0)	
Emergency	36 (21.6)	40 (33.3)	
Ultima ratio	3 (1.8)	2 (1.7)	
Type of CIED			
ICD	80 (47.9)	59 (49.2)	0.91
Pacemaker	86 (51.5)	61 (50.8)	1.00
Leads older than one year	126 (75.5)	106 (88.3)	0.05
Prior myocardial infarction	41 (24.7)	28 (23.4)	0.50
History of CAD			0.03
One-vessel CAD	22 (13.2)	19 (15.8)	
Two-vessel CAD	14 (8.4)	23 (19.2)	
Three-vessel CAD	34 (20.4)	24 (20.0)	
Unspecified	3 (1.8)	0 (0)	
No CAD	90 (53.9)	54 (45.0)	
Unknown	4 (2.4)	0 (0)	
NYHA			0.90
I	35 (21.0)	28 (23.3)	
II	49 (29.3)	34 (28.3)	
III	65 (38.9)	48 (40.0)	
IV	16 (9.6)	8 (6.7)	
Number of prior heart surgeries			0.28
1	36 (21.6)	17 (14.2)	
2	6 (3.6)	4 (3.3)	
3	2 (1.2)	2 (1.7)	
4	0 (0)	0 (0)	
5	3 (1.8)	0 (0)	

The values are displayed in n (%) or mean ± SD. CAD = coronary artery disease, CIED = cardiac implantable electronic device, COPD = chronic obstructive pulmonary disease, CVA = cerebrovascular accident, ICD = implantable cardioverter defibrillator, LVEF = left ventricular ejection fraction, NYHA = New York Heart Association, RRT = renal replacement therapy.

There were no statistically significant differences in initial symptoms between the groups (*p* = 0.43). The most common clinical presentation in both groups were pacemaker pocket infection and fever. Other symptoms, such as fatigue, poor appetite, weight loss, neurological symptoms, and dyspnea, had similarly low prevalence and did not differ significantly between groups.

Although the EuroSCORE II was similar between the groups, the CAG group showed significantly higher rates of lead vegetation, with 65.0% of patients in this group affected compared to 52.7% in the non-CAG group (*p* = 0.04); and was more likely to have a prior history of two-vessel CAD, with a prevalence of 19.2% compared to only 8.4% in the non-CAG group (*p* = 0.03).

### 3.2 Study outcomes

Table 2 shows a summary of the postoperative outcomes between the two groups. There was no significant difference in postoperative complications between the groups in most measured outcomes.

**Table 2:** Summary of outcomes.

	No angiography n = 167	angiography n = 120	p-Value
30-day mortality	16 (9.6)	11 (9.2)	1.00
Myocardial infarction	0 (0)	0 (0)	–
Bleeding	16 (9.6)	12 (10.0)	1.00
Acute renal failure	33 (19.8)	21 (17.7)	0.76
Surgical conversion	3 (1.8)	5 (4.2)	0.29
Reason for conversion			0.16
Severe lead adhesions	0 (0)	1 (20.0)	
Lead torn off	0 (0)	3 (60.0)	
Pulseless electrical activity	1 (33.3)	0 (0)	
CABG performed after conversion	0 (0)	0 (0)	–
Cerebrovascular event	5 (3.0)	2 (1.7)	0.48
Vascular rupture	0 (0)	0 (0)	–
Gastrointestinal complications	7 (4.2)	5 (4.1)	0.42
Pericardial effusion	6 (3.6)	3 (2.5)	0.89
Pleural effusion	72 (43.1)	64 (53.3)	0.32
Pneumothorax			0.38
Drainage	4 (2.4)	1 (0.8)	
Punction	0 (0)	1 (0.8)	
Conservative treatment	1 (0.6)	0 (0)	
Rhythm at discharge			0.27
Sinus rhythm	68 (40.7)	53 (44.2)	
Atrial fibrillation	37 (22.2)	23 (19.2)	
Atrioventricular block	2 (1.2)	0(0)	
Pacemaker rhythm	40 (24.0)	26 (21.7)	
Discharge with pacemaker	65 (38.9)	47 (39.2)	1.00
Discharge with ICD	32 (19.2)	25 (20.8)	0.77
Discharge with defibrillation west	19 (11.4)	24 (20.0)	0.05

The values are displayed in n (%). CABG = coronary artery bypass grafting, ICD = implantable cardioverter defibrillator.

The rates of 30-day mortality were nearly identical, with 9.6% in the non-CAG group compared to 9.2% in the CAG group (*p* = 1.00). Similarly, there were no occurrences of myocardial infarction in either group. There was no difference regarding bleeding events (p = 1.00) and acute renal failure rates (*p* = 0.76).

The surgical conversion rates were low in both groups, with 1.8% in the non-CAG group and 4.2% in the CAG group (*p* = 0.29). Importantly, no coronary artery bypass (CABG) procedures were performed after conversion in both groups. Finally, the discharge outcomes were consistent across the patient groups, with no significant differences observed in the rates of discharge with pacemaker (*p* = 1.00) or implantable cardioverter defibrillator (*p* = 0.77).

## 4 Discussion

Our analysis suggests that preoperative CAG does not significantly influence short-term outcomes after CIED explantation due to infection such as mortality and major postoperative complications.

Currently, there is no definitive guideline in the literature advocating for routine CAG before CIED explantation due to infection [10], [11]. Nonetheless, surgeons typically want to ensure that if they proceed with opening the sternum and potentially need to use the heart-lung machine, the presence of significant CAD will not interfere with the preoperative strategy or complicate the weaning from cardiopulmonary bypass. Although our numbers are small, the results suggest that routine CAG before CIED may not be required. This raises the possibility that, among the converted patients, potential targets for intervention were simply absent. The intention behind the sternotomy seems to be solely to manage potential bleeding due to an unexpected perforation. However, for appropriate assessment of this question, randomized studies are needed.

Potentially omitting CAG before CIED removal may be associated with a reduction in healthcare-related costs, including those related to personnel, procedural expenses, and overall hospital stay. In our analysis, none of the patients requiring CIED explantation underwent concomitant bypass surgery. This observation strongly suggests that routine preoperative CAG may not be necessary in all patients, particularly when the likelihood of significant CAD requiring simultaneous revascularization is low. The literature appears somewhat sparse on this topic, as there are no other major series that have investigated this hypothesis.

Furthermore, it may be argued that patients presenting with sepsis may indicate a more severe clinical condition. Septic patients are often hemodynamically unstable, making invasive procedures such as CAG riskier and less feasible. In these cases, clinicians may have prioritized immediate infection control and hemodynamic stabilization over additional diagnostic procedures. This suggests a potential treatment allocation bias [12], as sicker patients were likely managed differently, with a more conservative approach that avoided unnecessary procedural risks. Consequently, the decision to decline CAG in these cases may reflect clinical judgment tailored to the urgency of infection management rather than a standardized protocol. In any case, despite this possibility, the group without CAG did not perform worse than the CAG group, which lends further support to our conclusion. Taking all those facts into consideration patients scheduled for CIED explantation due to infection might benefit most from omitting preoperative CAG in order to avoid delays to surgery and to prevent additional complications, without compromising patient safety. That's why this study focused on this patient cohort. Our intention was not to extrapolate the results of this single-center cohort to patients undergoing CIED explantation for other indications, such as chronic pain syndromes or lead dislodgement.

Ultimately, given the inherent risks of performing CAG in critically ill patients, alternative non-invasive imaging such as coronary CT angiography (CCTA) may be a valuable option in some specific scenarios for the following reasons: [I] advances in CCTA now allow high-resolution delineation of coronary anatomy with excellent sensitivity and specificity for significant CAD [13], [14], [15], [16], [17]. Notably, results from the FAST-TRACK trial led by Dr. Serruys suggest that a CCTA-first strategy can safely and efficiently rule out obstructive disease in selected patients, supporting its use as a gatekeeper to invasive angiography in appropriate clinical contexts [18]; [II] CCTA can be acquired alongside routine chest CT scans used for surgical planning, which is particularly useful in re-operative settings; [III] CCTA is non-invasive and avoids procedural complications associated with catheterization, such as vascular injury, bleeding or arrhythmias, making it particularly suitable for critically ill or high-risk patients who may not tolerate invasive procedures [13], [14], [15], [16], [17]. However, CCTA is less reliable in patients with prior PCI due to limitations in accurately characterizing stented segments and complex lesions [19].

### 4.1 Study limitations

The retrospective nature of the study implies different possible biases due to residual cofounders. Additionally, an important limitation of this study is the lack of follow-up data, which significantly restricts the capacity to evaluate long-term outcomes in patients undergoing CIED explantation. Without longitudinal data, it is not possible to assess the durability of short-term findings or other long-term implications of untreated conditions. Finally, we do not know whether patients receiving CAG experienced any complications before surgery, because we were only able to evaluate peri-operative outcome.

## 5 Conclusion

The single-center experience suggests that preoperative CAG does not significantly influence short-term outcomes after CIED explantation due to infection. Systematic preoperative CAG may not be necessary for all patients, but randomized evidence is required to make this conclusion generalizable.

## CRediT authorship contribution statement

**Tulio Caldonazo:** Writing – review & editing, Writing – original draft, Visualization, Validation, Supervision, Software, Resources, Project administration, Methodology, Investigation, Funding acquisition, Formal analysis, Data curation, Conceptualization. **Holly Scheler:** Writing – review & editing, Writing – original draft, Methodology, Formal analysis, Data curation, Conceptualization. **Johannes Fischer:** Writing – review & editing, Writing – original draft, Methodology, Data curation, Conceptualization. **Hristo Kirov:** Writing – review & editing, Validation, Supervision, Software, Resources, Project administration, Methodology, Investigation, Formal analysis, Data curation, Conceptualization. **Murat Mukharyamov:** Writing – review & editing, Writing – original draft, Validation, Supervision, Resources, Methodology, Investigation, Conceptualization. **Stephanie Gräger:** Writing – review & editing, Validation, Supervision, Methodology, Formal analysis, Data curation, Conceptualization. **Angelique Runkel:** Writing – review & editing, Validation, Methodology, Investigation. **Sebastian Reinartz:** Writing – review & editing, Validation, Supervision, Methodology, Investigation, Conceptualization. **Mahmoud Diab:** Writing – review & editing, Validation, Supervision, Resources, Project administration, Data curation, Conceptualization. **Torsten Doenst:** Writing – review & editing, Writing – original draft, Validation, Supervision, Resources, Project administration, Methodology, Funding acquisition, Conceptualization.

## Funding

TC was funded by the 10.13039/501100001659Deutsche Forschungsgemeinschaft (DFG, German Research Foundation) Clinician Scientist Program OrganAge funding number 413668513, by the 10.13039/501100005971Deutsche Herzstiftung (DHS, German Heart Foundation) funding number S/03/23 and by the Interdisciplinary Center of Clinical Research of the Medical Faculty Jena.

## Declaration of competing interest

The authors declare the following financial interests/personal relationships which may be considered as potential competing interests: Tulio Caldonazo reports financial support was provided by German Heart Foundation. Tulio Caldonazo reports financial support was provided by German Research Foundation. If there are other authors, they declare that they have no known competing financial interests or personal relationships that could have appeared to influence the work reported in this paper.

## Data Availability

The data underlying this article are available under request.

## Glossary

CABG: coronary artery bypass grafting
CAD: coronary artery disease
CAG: coronary angiograms
CCTA: coronary CT angiography
CIED: cardiac implantable electronic device
IQR: interquartile range
